# Emergency Department Visits Within 90 Days of Elective Hand Surgery: A Healthcare Utilization Study

**DOI:** 10.1177/22925503251371057

**Published:** 2025-09-03

**Authors:** Katie Ross, Haley Glazebrook, Jo-Anne Douglas, Justin MacLellan, Emily M. Krauss

**Affiliations:** 1 Division of Plastic Surgery, Department of Surgery, 12361 Dalhousie University, Halifax, Canada; 2 Faculty of Medicine, 12361 Dalhousie University, Halifax, Canada; 3 Department of Surgery, 12361 Dalhousie University, Halifax, Canada

**Keywords:** Carpal tunnel release, trigger finger release, elective hand surgery, emergency department, healthcare utilization, Libération du canal carpien, décompression d’un doigt gâchette, chirurgie élective de la main, service des urgences, utilisation des ressources de santé.

## Abstract

**Introduction:** Emergency department (ED) visits following elective surgeries place significant strain on already overburdened healthcare systems. In Nova Scotia, carpal tunnel release (CTR) and trigger finger release (TFR) are the most common elective hand surgeries performed. This study sought to determine the rates, reasons, and risk factors associated with ED visits following elective outpatient hand surgery. **Methods:** Patients who underwent CTR or TFR between April 1, 2016 and March 31, 2022, and visited any Nova Scotia ED within 90 days of surgery were identified using provincial healthcare databases. A chart review was completed to explore ED timing, reasons for presentation, and predetermined systems-level factors. **Results:** During the retrospective period, 2690 patients underwent CTR and 1103 patients underwent TFR. For CTR, 159 patients (5.97%) presented to the ED within 90 days of surgery for surgery-specific concerns. The most common presentation was surgical site infection (2.16%), wound check (1.60%), and suture removal (1.04%). For TFR, 63 (5.71%) patients presented to the ED within 90 days. Similarly, surgical site infection was the most common presentation (2.63%) followed by suture removal (1.45%) and wound check (1.18%). The most frequent timing for ED presentation was days 13-15 (27.9%). **Conclusion:** In an evaluation of true healthcare utilization after elective surgery, ED visits for surgery-specific concerns after CTR and TFR were nearly 6%, far exceeding expected complication rates. As the presentations are manageable in an outpatient clinic or office setting, a mixed-methods patient-oriented intervention is planned to redirect this population away from the ED.

## Résumé

**Introduction :** Les visites aux services des urgences après des interventions chirurgicales programmées créent un fardeau significatif sur des systèmes de soins de santé déjà surchargés. En Nouvelle-Écosse, la libération du canal carpien (LCC) et la décompression d’un doigt gâchette (DDG) sont les deux opérations électives de la main les plus fréquentes. Cette étude a cherché à déterminer les fréquences, les raisons et les facteurs de risque associés aux visites aux services des urgences après chirurgie élective de la main chez des patients ambulatoires. **Méthodes :** Des patients ayant bénéficié d’une LCC ou d’une DDG entre le 1^er^ avril 2016 et le 31 mars 2022, s’étant rendu dans n’importe lequel des services d’urgences de Nouvelle-Écosse ont été identifiés à partir des bases de données provinciales de soins de santé. Une revue des dossiers a été menée pour explorer le moment relatif de la visite aux urgences, les raisons de la visite ainsi que des facteurs prédéterminés au niveau des systèmes. **Résultats :** Au cours de la période envisagée, 2690 patients ont subi une LCC et 1103 patients ont subi une DDG. Concernant la LCC, 159 patients (5,97%) sont allés aux urgences dans les 90 jours suivant l’intervention pour des préoccupations concernant spécifiquement la chirurgie. La présentation la plus fréquente était une infection du site chirurgical (2,16%), une vérification de la plaie (1,60%) et le retrait de sutures (1,04%). Concernant la DDG, 63 patients (5,71%) se sont présentés aux urgences dans les 90 jours. De même, une infection du site chirurgical était le motif de présentation le plus fréquent (2.63%), suivie du retrait de suture (1,45%) et de la vérification de la plaie (1,18%). Le moment le plus fréquent pour la visite aux services d’urgences se situait entre les jours 13 à 15 (27,9%). **Conclusion :** Dans une évaluation de la véritable utilisation des ressources de santé après chirurgie programmée, les visites aux services des urgences pour des préoccupations spécifiquement liées à la chirurgie après LLC et DDG avoisinaient 6%, dépassant de loin les taux de complications attendus. Dans la mesure où les présentations sont gérables dans une clinique de soins ambulatoires ou dans un cadre de bureau, une intervention orientée sur les patients mélangeant plusieurs méthodes est prévue pour rediriger cette population hors des services d’urgences.

## Introduction

Unplanned visits to the emergency department (ED) following surgery place a significant strain on the healthcare system and contribute to rising costs.^1^ While 30-day hospital readmission rates following surgery are commonly used as an indicator of quality of care, ED visits are less frequently studied.^1-3^ Particularly in scenarios where postoperative readmissions are low, evaluating ED utilization may offer a more accurate measure of healthcare usage and provide valuable insights into modifiable factors that could help prevent these rebounds.^4,5^

Carpal tunnel and trigger finger release (TFR) are among the most common elective outpatient hand procedures performed.^6,7^ Studies have investigated inpatient readmission rates after upper extremity and hand surgeries, but they often overlook ED visits where patients received care and were discharged home.^8,9^ Three different studies report similar ED rebound rates within 30 days, reporting 2.7%,^6^ 4.4%,^3^ and 3.0%.^2^ One study reported 90-day ED rebound rates and found that 3.8% of patients returned to the ED in that timeframe.^7^ As the volume of ambulatory hand surgery grows, so does the importance of identifying factors that contribute to unplanned ED visits and the associated healthcare costs.^10-12^

The purpose of this study was to examine the rates and reasons for unplanned ED visits within 90 days of elective outpatient carpal tunnel release (CTR) and TFR surgery. This study evaluates ED visits across an entire province, after surgery was performed at one of three hospitals in a centralized geographic area, which represents the major referral center for the province. Typically, patients would have surgery and then be scheduled to follow-up with the same surgeon 2 weeks postoperatively (either in person or virtually during the COVID-19 pandemic). Through a quality improvement lens, we sought to uncover areas for improvement in patient care, and education to reduce healthcare costs and improve patient experiences by minimizing unnecessary post-surgery ED visits outside of routine follow-up appointments.

## Methods

### Study Design and Population

This retrospective study included patients who underwent elective CTR or TFR surgery at three hospitals within the Nova Scotia Health (NSH) Central Zone between April 1, 2016 and March 31, 2022, in Nova Scotia, Canada. Eligible participants with a valid provincial health card and who had an ED visit in Nova Scotia within 90 days of postoperative discharge following their primary surgery were included for analysis. For patients who underwent bilateral CTR or TFR within 90 days, only the first surgery was considered for inclusion.

### Data Collection

Patients were identified using NSH administrative data sets: Discharge Abstract Database (DAD) and the National Ambulatory Care Reporting System (NACRS). Patients who had a primary procedure Canadian Classification of Health Intervention code of “1.BN.72.LA - Release, nerve(s) of forearm and wrist using open approach” or “1.UT.72.LA, Release, flexor tendons of finger [excludes thumb] using open approach” were included. Of note, “1.BN.72.DA - Release, nerve(s) of forearm and wrist -using endoscopic approach” was not included in the dataset as endoscopic CTR is not performed in this province.

Patient demographic (age, sex, patient residence urban/rural) and surgical descriptive variables (hospital, surgeon, date of surgery) were also obtained through DAD and NACRS. Urban/rural status was determined from the second digit of patient postal codes, where a zero as the second digit of the postal code represented rural.

ED visits were identified from NSH ED Provincial datasets Emergency Department Information System, System for Tracking and Analysis of Emergency Room, and Meditech to provide ED descriptive variables (ED site, ED length of stay, days from surgical discharge, and broad description of reason for ED visit based on triage). Anonymized information and study data were collected and managed using REDCap electronic data capture tools hosted at NSH for further analysis. Chart reviews of ED visits provided surgical/medical categorization of the reason for the visit as well as specific treatment(s) received during the ED visit. We sought to characterize the reasons for these ED visits, identify potentially avoidable encounters, and assess the patient demographic and visit features that may predispose patients to return to the ED following elective hand surgery.

### Statistical Analysis

The ratio of patients who presented at least once to any NSH ED within 90 days of their first CTR or TFR surgery, within the 6-year study period, was calculated. Descriptive statistics including frequencies, means, ranges, and standard deviation and were calculated using SAS software (Version 9.4 SAS System for Windows Copyright ^©^ 2016 SAS Institute Inc., Cary, NC, USA).

### Ethics

NSH REB exemption (1028137) was obtained citing this project as a Quality Improvement Project with Privacy and Quality Improvement division approval at NSH.

## Results

Between April 1, 2016 and March 31, 2022, 2690 patients had open CTR and 1103 patients had open TFR performed in a central zone hospital facility and were included in the study. Nineteen surgeons performed these hand surgeries, including plastic surgeons (n = 10), orthopedic surgeons (n = 7), and neurosurgeons (n = 2). In total, 625 of patients undergoing either CTR or TFR presented to the ED within 90 days of hand surgery. Of these, 448 were CTR patients (16.7% of all CTR patients) and 177 were TFR patients (16.0% of TFR patients). For the CTR population, 159 patients (35.5%) presented with surgical concerns and 289 (64.5%) presented with unrelated medical concerns. For the TFR group, 63 patients (35.6%) had surgical concerns and 114 (64.4%) had unrelated medical concerns. Analysis of medical concerns for ED visits found that none of the visits coded as medical were related to the surgery (ie, all visits were for medical conditions unrelated to their hand surgery that happened to occur within the 90-day post-surgical data capture window). When surgical reasons for ED visits were exclusively examined, 159/2690 (5.97%) CTR patients and 63/1103 (5.71%) TFR patients returned to the ED within 90 days of surgery for concerns specifically related to the surgery (combined 222/3793 or 5.85% ED return rate). TFR patients presenting to the ED over 90 days were slightly older than CTR patients over the same timeframe, with mean ages of 60.9 (± 13.2) and 55.9 (± 16.5) years, respectfully (Tables 1 and 2).

**Table 1.** Characteristics and Timing of ED Visits After CTR for Assessment for Surgery-Specific Concerns.

Characteristic	Timing of ED visit			
	1-7 days	8-30 days	31-90 days	1-90 days (Total)
Total ED visits, n (%)	39 (24.5%)	109 (68.5%)	11 (6.9%)	159 (100%)
Age in years, mean (SD)	56.5 (18.9)	55.8 (15.3)	54.6 (18.9)	55.9 (16.5)
Sex				
Female, n (%)	18 (46.1%)	66 (60.5%)	8 (72.7%)	92 (57.9%)
Male, n (%)	21 (53.9%)	43 (39.5%)	3 (27.3%)	67 (42.1%)
Patient Geography				
Rural, n (%)	12 (30.8%)	41 (37.6%)	3 (27.3%)	56 (35.2%)
Urban, n (%)	27 (69.2%)	68 (62.4%)	8 (72.7%)	103 (64.8%)
Primary care provider				
Has provider, n (%)				145 (91.2%)
Has no provider, n (%)				14 (8.8%)
Hours in ED, mean (SD)	2.1 (1.7)	2.3 (2.0)	2.7 (1.7)	2.3 (1.9)

ED, emergency department; CTR, carpal tunnel release.

**Table 2.** Characteristics and Timing of ED Visits After TFR for Assessment of Surgery-Specific Concerns.

Characteristic	Timing of ED visit			
	1-7 days	8-30 days	31-90 days	1-90 days (Total)
Total ED visits, n (%)	12 (19.0%)	45 (71.4%)	6 (9.5%)	63 (100%)
Age in years, mean (SD)	61.2 (9.13)	60.1 (12.9)	60.9 (14.4)	60.9 (13.2)
Sex				
Female, n (%)	6 (50%)	24 (53.3%)	3 (50%)	33 (52.4%)
Male, n (%)	6 (50%)	21 (46.7%)	3 (50%)	30 (47.6%)
Patient Geography				
Rural, n (%)	5 (41.7%)	14 (31.1%)	2 (33.3%)	21 (33.3%)
Urban, n (%)	7 (58.3%)	31 (68.9%)	4 (66.7%)	42 (66.7%)
Primary care provider				
Has provider, n (%)				57 (90.5%)
Has no provider, n (%)				6 (9.5%)
Hours in ED, mean (SD)	2.9 (2.0)	1.9 (1.6)	2.5 (1.2)	2.2 (1.7)

ED: emergency department; TFR, trigger finger release.

**Figure 1.**
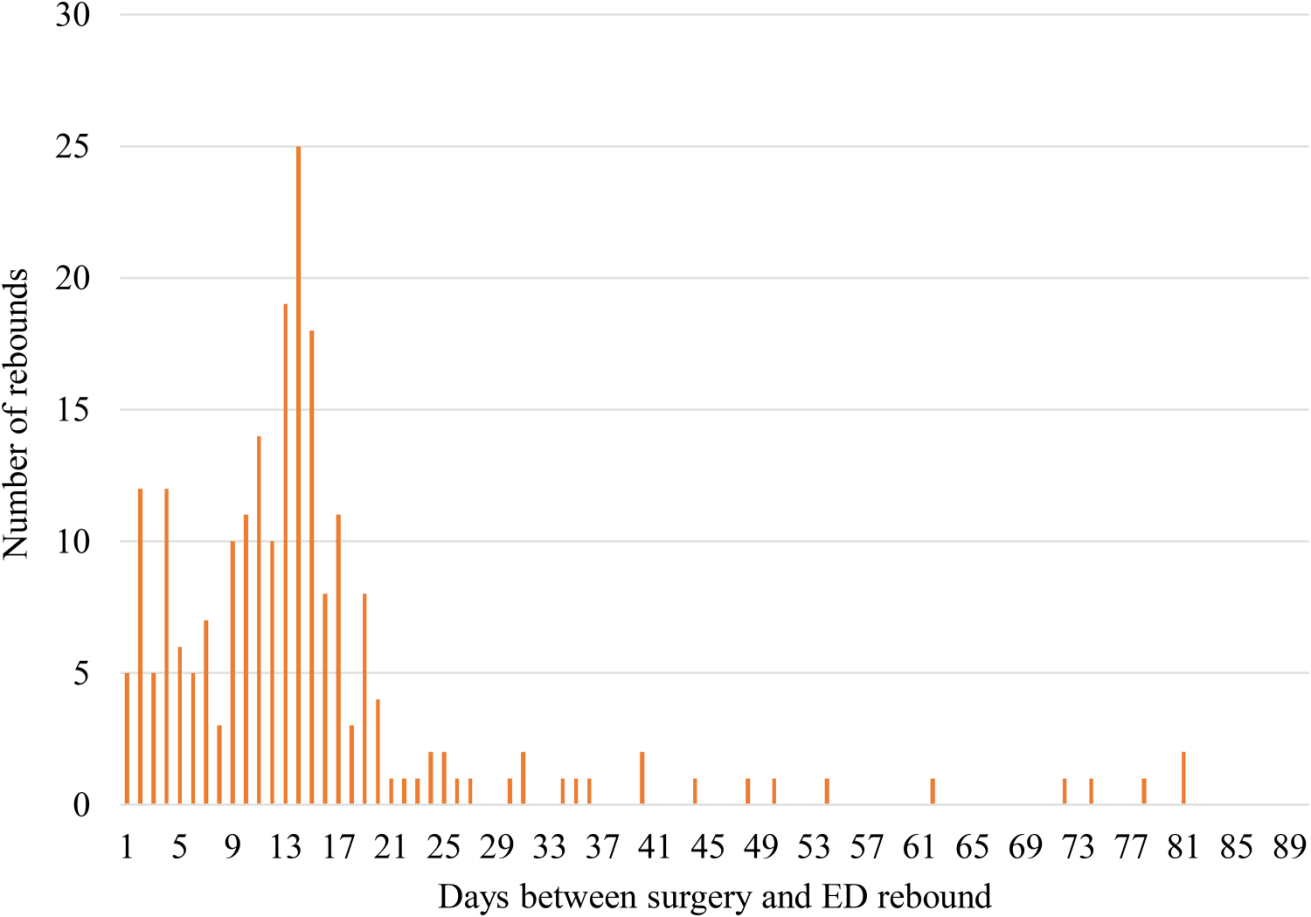
Frequency of ED visits for surgical concerns by days after hand surgery (1-90 days). ED, emergency department.

Although hand surgery was performed at one of three hospitals in a centralized geographic area (NSH Central Zone), patients presented to 25 different EDs across the province in four zones representing a geographic area of 55,283 km^2^ (34,330 miles^2^). No surgeries were performed in a private care setting. The timing of ED visits following CTR and TFR emphasizes the early postoperative period as a critical window. For CTR patients, nearly a quarter (n = 39, 24.5%) presented to the ED within the first week: 70.4% (n = 112) presented within the first 15 days; and 93.0% (n = 148) within the first 30 days (Table 1). Only 6.9% (n = 11) of patients presented between days 31-90 for surgery-specific concerns (Table 1). For TFR, 19.0% (n = 12) of patients presented within the first week, 79.4% (n = 50) within the first 15 days, and 90.5% within 30 days (Table 2). Similarly, only 9.5% (n = 6) of patients presented between days 31-90 (Table 2). Of note, postoperative day 13, 14, and 15 had the highest rates of return (representing 27.9% of patients; Figure 1). Patients receiving either surgery had an average ED visit time of 2.2 h (Table 3).

**Table 3.** Characteristics and Timing of ED Visits After Hand Surgery (CTR and TFR) for Assessment of Surgery-Specific Concerns.

Characteristic	Timing of ED visit			
	1-7 days	8-30 days	31-90 days	1-90 days (Total)
Total ED visits, n (%)	51 (23.0%)	154 (69.4%)	17 (7.7%)	222 (100%)
Age in years, mean (std)	58.9 (14.0)	58.3 (14.1)	57.8 (16.6)	58.4 (14.8)
Sex				
Female, n (%)	24 (47.0%)	90 (58.4%)	11 (64.7%)	125 (56.3%)
Male, n (%)	27 (53.0%)	64 (41.6%)	6 (35.3%)	97 (43.7%)
Patient Geography				
Rural, n (%)	16 (31.4%)	56 (36.4%)	5 (29.4%)	77 (34.7%)
Urban, n (%)	35 (68.6%)	98 (63.6%)	12 (70.6%)	145 (65.3%)
Primary care provider				
Has provider, n (%)				203 (91.4%)
Has no provider, n (%)				19 (8.6%)
Hours in ED, mean (SD)	2.2 (1.6)	2.1 (1.9)	2.6 (1.8)	2.2 (1.9)

ED: emergency department; CTR, carpal tunnel release; TFR, trigger finger release.

**Figure 2.**
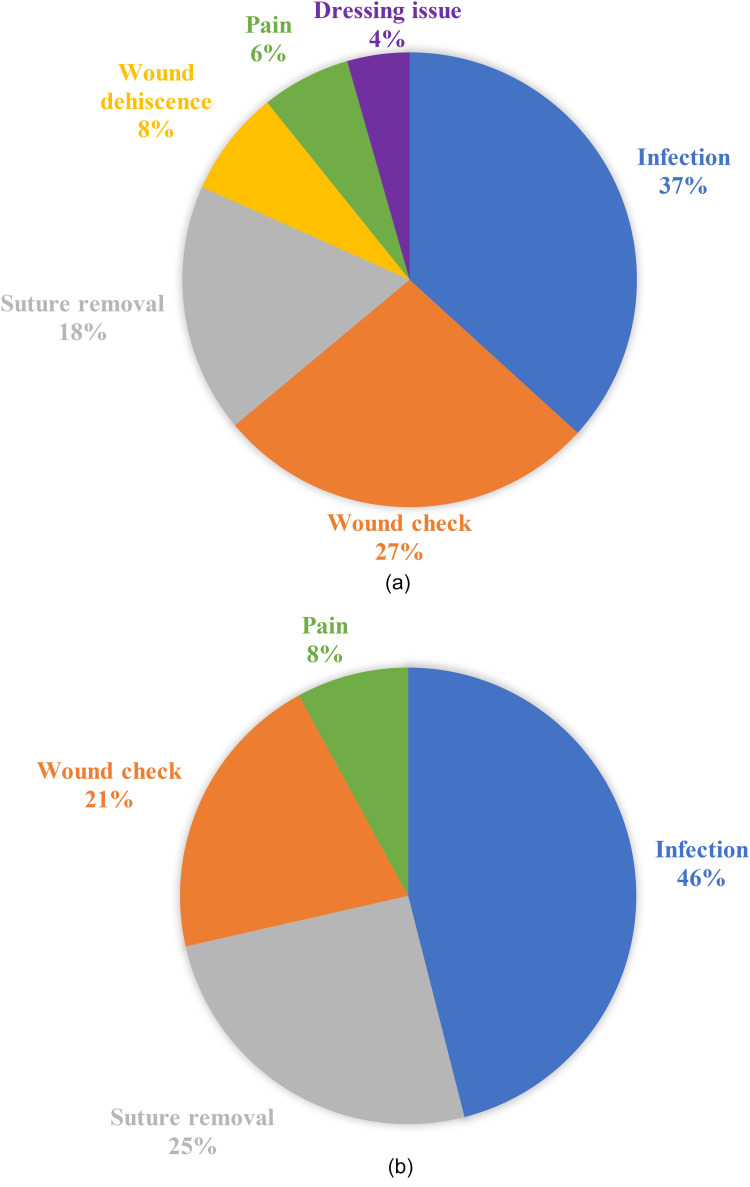
a) Pie chart showing the main surgical reasons for ED visit within 90 days of CTR. b) Pie chart showing the main surgical reasons for ED visit within 90 days of TFR. ED, emergency department; CTR, carpal tunnel release; TFR, trigger finger release.

No patients who presented with surgical concerns required admission to hospital for treatment, suggesting these patients could have been managed outside the ED to reduce the burden on emergency care facilities for non-urgent concerns. For CTR, the most common reasons for surgical presentation were surgical site infection (n = 58, 36.5%), wound check (n = 44, 27.0%), suture removal (n = 28, 17.6%), wound dehiscence (n = 12, 7.5%) pain (n = 10, 6.3%), and dressing/splint issues (n = 7, 4.4%) (Figure 2a). For TFR, the most common reasons were surgical site infection (n = 29, 46.0%), suture removal (n = 16, 25.4%), wound check (n = 13, 20.6%), and pain (n = 5, 7.9%) (Figure 2b). When reported in relation to the total number of hand procedures performed (n = 3793), the most common reasons for presentation were surgical site infection (n = 87/3793, 2.29%), wound check (n = 57/3793, 1.50%), suture removal (n = 44/3793, 1.16%), and pain (n = 15/3793, 0.40%). Importantly, surgical site infection was defined as prescription of antibiotics by the ED physician and not as positive bacterial culture results. Wound checks were defined as instances where the patient had a concern about their wound where no antibiotics were prescribed, and the concern did not fall into another category such as pain, suture removal, or wound dehiscence.

When evaluating for factors that may predict return to ED, data on primary care status was collected to determine whether our patients presenting to the ED did not have a primary care practitioner, as increasing access to primary care is a current provincial target for healthcare. We hypothesized that perhaps lack of access to primary care may be driving higher utilization of the ED in this surgical population. However, we found that over 91% of patients were listed as having a primary care provider (family physician or nurse practitioner; Table 3). Based on postal code locations, roughly two thirds (65.3%, n = 145) of patients resided in urban locations based the Statistics Canada's population counts for geographic locations (Table 3). In alignment with this proportion, twice as many urban-residing patients as rural patients presented to an ED at any given timepoint (Table 3). Our data did not capture further patient demographics based on geography.

## Discussion

This study included 3793 patients who underwent a CTR or TFR in Nova Scotia at one of three centralized hospitals. We investigated the number of patients who returned to the ED for surgical concerns within 90 days of surgery and identified a 5.85% rebound rate. Previous studies on ED use following elective outpatient hand and upper extremity surgery have ranged from 2.7% to 4.4%.^2,3,6,7^ This study is unique in that it analyzes data on a province-wide level and captures ED visits that may otherwise have not been reported to the primary surgeon. Our findings illustrate an ED visit rate higher than the expected rebound rate in elective hand surgery.

The rate of return to ED in this study is much higher than anticipated based on surgeon perspectives and what was inferred from postoperative phone calls and visits to our office. The typical patient had their outpatient hand surgery performed and then had a scheduled 2 week follow-up appointment with the surgeon. The majority of follow-up appointments were in person with a small number performed virtually either during the COVID-19 pandemic or at the patient's request. If complications occurred, patients were instructed to contact the surgeon's office for an urgent appointment. Patients were only instructed to present to the ED if they had an emergency or felt systemically unwell. This high rebound number illustrates a disconnect between the true utilization of the healthcare system by patients after minor surgery and the information collected by only assessing surgeon office visits. Patients had surgery performed at one of three centrally located hospitals, yet they presented to 25 different EDs across the province with a geographic area of 55,283 km^2^ (34,330 miles^2^). The geographic dispersion of patients highlights potential barriers in accessing the surgeon for timely follow-up care particularly for rural patients; however, this is only part of the picture as 65.3% of patients seeking the ED after elective hand surgery lived in urban areas. Exploring technology solutions to improve patient access to their surgeons (scheduled telehealth services, secure messaging platforms, or phone appointments) may provide patients from remote locations with enhanced care following their hand surgery. Additionally, patients listed as having a family physician may not be accurate. The health system does not automatically update when a family physician is not available (for example is on leave, retires, or moves away) so these patients could be wrongly identified as having a family physician. As well, a currently overburdened primary care system has resulted in common delays for patient appointments, affecting patient access to physician assessment for urgent concerns, like a surgical site infection.^13^

Days 13-15 had the highest rate of return with 27.9% of patients returning on those 3 days postoperatively, indicating an important time frame to target for reducing ED visits. This is a time when suture removal is typically performed. During the period included in this study, most patients had non-absorbable sutures and were seen in clinic for suture removal at 2 weeks. Some surgeons’ practices were to have patients see their family physician for suture removal and follow-up with their surgeon at 4 weeks. If they were unable to see their family care provider, they were asked to contact the plastic surgery clinic. It is clear from the number of patients attending the ED for suture removal that surgeon-specific practices can drive a small increase in ED utilization, which has since been addressed. If patients had absorbable sutures, they still had a scheduled follow-up at 2 weeks as this is a timeframe when mild foreign body reactions and hyperemia can be expected. This can be mistaken as signs of infection if patients are not counseled appropriately. Regardless of these standard practices, patients are still utilizing the ED inappropriately for suture removal and wound checks. An important consideration of this paper is the challenge posed by the centralized healthcare services in NS. Long travel distances and extended wait times to see specialists often hinder timely care. Some patients may have no choice but to visit the ED when issues arise after surgery and outside of regular service hours.^14^ This underscores the need to streamline postoperative patients away from the ED to ensure that only those with no other options seek ED services.

The most common reasons for presentation to the ED were surgical site infections (2.29%) and wound checks (1.50%), representing 64.9% of surgical ED rebound visits and overall complication rate of 3.79%. Surgical site infection after hand surgery performed under local anesthesia is low in the published literature, reported as low as 0.4% by LeBlanc et al in a comparable multicenter Canadian patient population.^15^ Recently, Sandefur et al investigated the rate of surgical site infection after CTR. They found the infection rate was heavily influenced by how infections are defined in patient charts. Infection rate defined as the prescription of an oral antibiotic was found to be 8.9% versus 2.3% for definition of infection by the Centers for Disease Control and Prevention and 0.4% for infection that required reoperation.^16^ In our ED system, “wound check” as a visit reason generally referred to patients seeking care for a suspected infection but who are not diagnosed with wound infection after physician assessment. As well, providing antibiotics to a patient presenting to the ED with wound concerns is commonly classified as a surgical site infection in hospital charting. The surgical site infection rate from the ED rebound data in this study is five times that of the LeBlanc et al study, either representing a higher infection rate in our hospital system or misdiagnosis of postoperative infection using ill-defined criteria and assessment by ED physicians. This may indicate an opportunity for better patient education on the expected healing course after CTR and TFR, specific signs of infection, and a more appropriate redirection of these patients to their surgeon's offices for expert assessment and management.

Pain, which accounted for 6.7% of all surgical visits, was notably less common than reported in similar studies conducted in other healthcare settings. Menendez and Ring reported a 3% 30-day ED visit rate for carpal tunnel and TFRs with pain and wound complications being the predominant causes.^2^ Similarly, Sivasundaram et al reported a 4.4% ED return rate within 30 days, with pain being the leading cause, while Benage et al identified a slightly lower ED visit rate of 2.7% within 30 days, with common reasons including pain, swelling, and infection concerns.^3,6^ Another study by Nasser and colleagues examining 30-day ED utilization after distal radius fracture also found a postoperative ED return rate of 2% with pain as the primary complaint; however, this does not include patients who received closed treatment for the same injury, who presented to the ED at a higher rate.^17^ Interestingly, that study also found significant differences in ED utilization based on geography; however, they were able to subdivide their patients into distinct regions, where we were only able to identify urban/rural status. Shetty et al assessed patient-reported data (PRD) and preventable ED visits within 90 days of outpatient hand surgery. They reported an ED rebound rate of 3.8% in their population and found that worse pre-operative PRD scores (Patient-Reported Outcomes Measurement Information System, PROMIS, upper extremity, and Pain Interference scales) were associated with higher ED rebound rates, potentially identifying patients that may benefit from additional support.^7^

**Figure 3.**
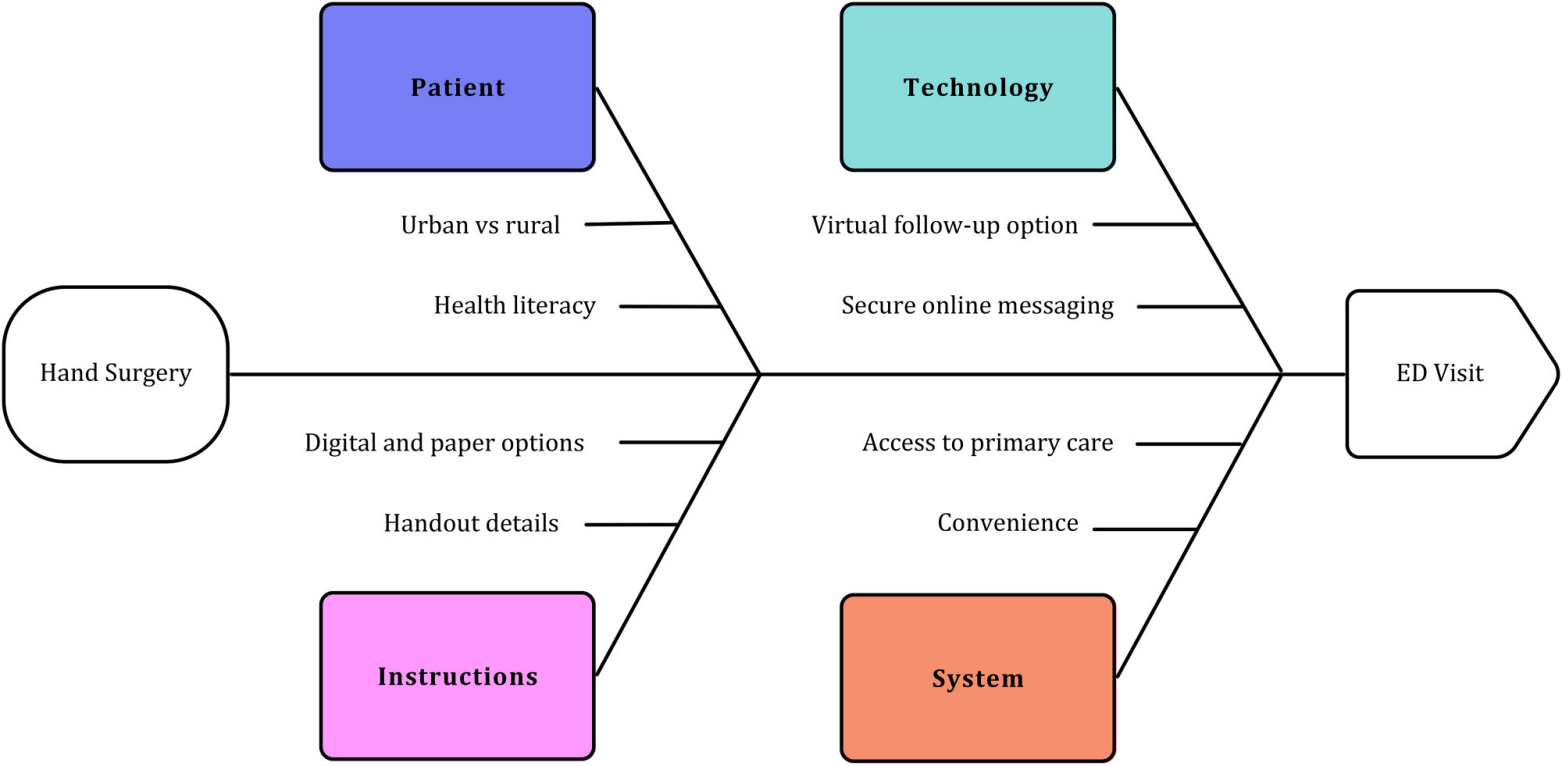
Fishbone diagram of potential reasons and factors contributing to ED visits following hand surgery (CTR and TFR). Although patient factors such as health literacy and geographic distribution cannot be changed, considering various solutions such as improving patient instructions (detail, format, delivery), using technological solutions (virtual visits, secure online messaging), and influencing system factors (improving access to primary care, improving the convenience of accessing and contacting the operating surgeon) are all potential targets for interventions to reduce ED utilization for non-urgent issues in hand surgery patients. ED, emergency department; CTR, carpal tunnel release; TFR, trigger finger release.

Given that all surgical postoperative presentations were discharged home and could have been managed by the surgeon, patient reasons for ED presentation need to be assessed. In our current study, over 91% of patients had access to a primary care provider, thus lack primary care access does not appear to be a driver of high ED utilization in this post-surgical population. Hanson et al investigated the decision-making process behind why patients choose the ED when urgent care is not needed. They found multiple factors including the perceived severity of condition and convenience.^18^ If patients believe their condition is serious, often determined by the severity of pain or history of similar complications in the past, they are likely to present for care. Further, if the ED is the most convenient location for care, patients will often choose the ED over alternatives. Convenience can include hours of operation, travel distance, and familiarity.^18^ Similarly, Uscher-Pines et al performed a systematic review of studies assessing why patients visit the ED for non-urgent conditions. They also found that convenience of the ED and negative perceptions about alternatives play a role in driving non-urgent ED use.^19^ To reduce ED usage for non-urgent concerns following outpatient hand surgery, ensuring that patients are well-educated on the expected course of recovery, and improving the convenience for patients to access their surgeon (or their family physician) versus the ED are important first targets for intervention. Providing patients with clear instructions about wound care and pain management that are appropriate for patient literacy levels and offering paper and digital options would be beneficial. Scheduled follow-up or the reliability of surgeons and office staff to be contacted for short notice appointments could also be addressed. Leveraging telehealth services, such as virtual wound assessments or secure messaging with images, may offer a practical and convenient alternative for rural patients (Figure 3).

The strengths of this study include its comprehensive review of ED visits across a large geographically diverse cohort within a single healthcare system, providing insight into regional healthcare utilization patterns. As this is a Canadian study in a single province with single-payer health insurance, without the availability of private delivery of outpatient hand surgery, the information included in the study represents true usage patterns across a large geographic area without the possibility of missing healthcare encounters outside of network or within a different healthcare delivery system. The inclusion of patient factors, timing, and reasons for ED visits allows for targeted recommendations to improve postoperative care. The inclusion of a 90-day follow-up period provides a more comprehensive understanding of postoperative complications than previous studies. Several limitations warrant consideration. Our study design is descriptive, and data was limited to analysis of historical charts. The retrospective nature lacks details about patient perspectives for ED presentation (belief that concern is a true emergency, unable to access surgeon or other non-urgent healthcare facility, etc). We also categorized patients as “wound check” if they had concerns about their wound but did not receive antibiotics and “wound infection” if they had concerns about their wound and received antibiotics, reflecting vague criteria for wound assessments and the poor documentation of wound physical examination leading to the diagnosis of a surgical site infection. The study did not assess patient-reported outcomes or satisfaction, which could provide a more nuanced understanding of factors driving ED utilization. To avoid counting the same patient twice, additional ED visits by the same patient were excluded. While this avoided selection bias in our sample, the true ED utilization by this patient population may be higher than reported. This cohort is from one Canadian province with an integrated public healthcare system, limiting generalizability to other models of healthcare delivery.

## Conclusion

This study illustrates healthcare utilization after common elective outpatient hand surgery and illustrates a higher rate of ED visits than expected. In an overburdened public healthcare system, reducing unnecessary ED visits can have significant effects on access to timely care for emergency presentations. This study provides preliminary data on potential targets for hand surgeons, specifically by improving patient education on wound healing and signs of surgical site infection, improving access to assessments between 13 and 15 days after surgery, and improving the convenience of accessing the surgeon, to avoid unnecessary ED visits to reduce system burden while enhancing care of hand surgery patients.
